# Medical Consultation and HIV Testing After AI-Based Symptom Check: Retrospective Cohort Study

**DOI:** 10.2196/90257

**Published:** 2026-06-18

**Authors:** Nao Taguchi, Kunihiro Hirahara, Keisuke Harada, Keisuke Orimo, KuanYeh Lee, Kota Iwahashi, Akifumi Imamura

**Affiliations:** 1 Gilead Sciences K.K., Chiyoda-ku, Tokyo, Japan; 2 Ubie Inc, Chuo-ku, Tokyo, Japan; 3 Nonprofit Organization akta, Shinjuku-ku, Tokyo, Japan; 4 Tokyo Metropolitan Cancer and Infectious Diseases Center Komagome Hospital, 3-18-22 Hon-komagome, Bunkyo-ku, Tokyo, 113-8677, Japan, 81 3-3823-2101

**Keywords:** HIV infections, sexually transmitted diseases, artificial intelligence, AI, symptom assessment, delayed diagnosis, patient acceptance of health care, health knowledge, attitudes, practice, Japan, sexually transmitted infection

## Abstract

**Background:**

Promoting early HIV testing and patient detection is an important public health goal. In Japan, approximately 30% of the population is diagnosed with AIDS. Several studies have investigated the challenges related to HIV diagnosis; however, there are limitations in understanding the characteristics and barriers faced by individuals who are at high risk of HIV but have not yet been tested or have not sought medical consultation.

**Objective:**

This study aimed to examine the factors associated with medical consultation and HIV-testing behaviors, explore the reasons for not undergoing HIV testing, and evaluate the effectiveness of HIV-related awareness efforts among respondents to a revisit survey conducted via an artificial intelligence (AI)–based symptom search engine.

**Methods:**

This retrospective cohort study used data obtained from the AI-based symptom search engine, Ubie. Episodes involving individuals who used the AI-based symptom checker to search for their symptoms, which were subsequently suggested as HIV/AIDS/sexually transmitted infection (STI)–related conditions, were included. Those who answered the first and revisit survey questionnaires were included in the analysis. Multivariable logistic regression analyses were conducted to explore the factors associated with medical consultation in both the overall suggested HIV/AIDS/STI–related condition group and the suggested STI-related condition subgroup. Factors associated with HIV testing in individuals who underwent medical consultations were also explored using multivariable logistic regression analysis. The reasons for not undergoing HIV testing and the future intention to undergo testing were described.

**Results:**

The number of eligible episodes was 424,893 for 332,976 individuals. Of these, medical consultations were performed in 105,365 cases and HIV testing in 394 cases. Compared with individuals in their 20s, older age groups were associated with a higher tendency to seek medical consultations. The provision of awareness information through the AI-based symptom checker was associated with medical consultation behavior, and 29% (280/964) of people who initially had no intention of undergoing HIV testing responded that they would undergo HIV testing after using the AI-based symptom checker. Compared with the internal medicine department, the gynecology department was significantly associated with HIV testing; however, the HIV testing rates were low in the suggested STI-related condition subgroup across major departments.

**Conclusions:**

These results suggest that HIV-related information delivered via an AI-based symptom checker may raise awareness or consideration of medical consultation among individuals actively searching for symptoms potentially associated with HIV. To further promote HIV testing, it may be necessary to refine the content and delivery of educational materials and enhance HIV testing literacy among physicians who encounter patients with STIs.

## Introduction

The global epidemic of HIV remains a significant public health issue. In 2024, an estimated 40.8 million people were living with HIV, and there were approximately 1.3 million new HIV infections [1]. The Joint United Nations Program on HIV/AIDS has set the “95-95-95” targets for 2025 with the aims of (1) diagnosing 95% of individuals with HIV, (2) ensuring 95% of diagnosed individuals receive treatment, and (3) achieving viral suppression in 95% of treated individuals [2]. Of these targets, achieving the first “95” target—diagnosing 95% of individuals with HIV—is crucial, as it represents the entry point into the HIV care continuum. The importance of early awareness of one’s HIV status has also been emphasized in the Global AIDS Strategy 2021-2026 [3]. Because HIV infection itself does not cause specific symptoms, it is essential to connect people at risk of HIV infection with testing services to improve diagnosis rates before the onset of AIDS.

In Japan, approximately 30% of the population has been first diagnosed at the AIDS stage over the past decade [4,5]. The widespread availability of HIV testing has been facilitated by government- and community-based efforts, including anonymous testing at public health centers and promotional activities conducted by community-based organizations. However, achieving the first “95” target of diagnosis remains a challenge. Although the HIV diagnosis rate steadily increased before the COVID-19 pandemic, a significant decline in both testing and consultation numbers was observed in 2020, the first year of the pandemic. HIV testing numbers have started to increase as the pandemic gradually recedes, and the diagnosis rate reached 89.3% by 2022 [6]. However, HIV testing numbers remain below prepandemic levels [4]. Furthermore, a previous study reported that only approximately 5% of patients who tested for syphilis underwent HIV testing, indicating missed opportunities for early HIV detection [7]. These situations suggest that additional approaches are required to achieve the first “95” target of diagnosis.

Understanding the factors and barriers associated with HIV testing and consultation behaviors is important for improving diagnosis rates. Previous studies in Japan have identified several factors associated with the timing of HIV diagnosis. For instance, a study using data from the Japanese Drug Resistance HIV-1 Surveillance Network found that factors such as age, heterosexual transmission, living outside Tokyo, and hepatitis C virus coinfection were associated with late diagnosis between 2003 and 2019 [8]. Similarly, a retrospective cohort study of Japanese HIV care hospitals found that voluntary testing and being a man who has sex with men were associated with early diagnosis [9]. Furthermore, a study from Japan’s largest HIV referral center reported that the reasons for diagnostic tests in newly infected individuals included existing diseases, such as AIDS and sexually transmitted infections (STIs), voluntary testing, and routine presurgery or on-admission screening [10]. Regional differences have also been observed in Japan, with a higher proportion of undiagnosed HIV cases in nonmetropolitan areas [11].

Although previous studies have provided valuable insights into the experiences and challenges of individuals diagnosed with HIV, they have primarily focused on populations already engaged by the health care system. Expanding the scope of inquiry to include individuals at high risk of HIV who have not yet undergone testing or sought medical consultation offers an important opportunity to deepen our understanding of the diverse factors influencing testing behaviors. Recent reports suggest that novel approaches using digital technologies [12], artificial intelligence (AI), and machine learning can efficiently identify at-risk individuals [13], thereby potentially addressing this gap. By leveraging data from AI- and digital-based services that target high-risk populations, researchers can identify and study previously inaccessible high-risk groups.

In this study, we targeted individuals who were using the widely used web service of an AI-based symptom checker in Japan and identified them as the study population who reported symptoms potentially associated with the risk of HIV and responded to the revisit survey. The aim of this study was to examine the factors associated with medical consultation and HIV testing behaviors among these revisit survey respondents to better understand the challenges of HIV testing. We also explored the reasons for not undergoing HIV testing and evaluated the effectiveness of HIV-related awareness efforts.

## Methods

### Study Design and Data Source

This retrospective cohort study used data obtained from an AI-based symptom search engine, Ubie [14]. The symptom checker evaluated in this study is publicly available. Most users access it by conducting ordinary web searches for their symptoms (eg, Google or other search engines), which directly lead them to the publicly accessible Ubie symptom checker page. This service allows users to answer an AI-optimized adaptive question flow in a step-by-step format based on their symptoms and provides information on related conditions, recommended actions, appropriate medical departments, and health care facilities. The AI medical interview assistance system alternates question selection and identifies relevant diseases using a flowchart model regarding symptoms and diseases based on information from tens of thousands of medical reports. This service has approximately 10 million monthly users. Individuals experiencing symptoms access the symptom search engine through web searches or Ubie applications. In this AI-based symptom search engine, individuals who agreed to the service’s privacy policy responded to questions from the AI-based symptom checker. The results displayed potentially related conditions, along with the recommended health care facilities (Figure S1 in Multimedia Appendix 1). When individuals revisit the search engine after completing the initial questionnaire, they are asked to answer follow-up questionnaires regarding the health care consultations conducted since their initial visit.

Three types of data sources were used. First, survey data were collected from individuals who visited Ubie’s website, responded to symptom-related questions, and received presentations of potentially related conditions. The data sources included information on age, sex, comorbidities, treatment status, current symptoms, pain scores, and possible conditions. Second, revisit survey data were gathered, which included responses from individuals who revisited the website after completing the first survey, including data on their medical consultations and testing behaviors after the first survey. The revisit survey responses were automatically linked to the latest symptom check episode using an anonymized user identifier assigned within the platform. This data source included information on whether a medical examination or test was carried out, the type and area of the medical facility and department visited for consultation or testing, diagnostic results, and whether HIV testing was conducted. Third, HIV-specific banner questionnaire data were collected, which included responses to HIV-specific questions from individuals who completed a questionnaire accessed via banner advertisements on Ubie’s website during the first survey. This data source included information on reasons for not undergoing HIV testing and future intentions for HIV testing. The study period was from October 19, 2022, to December 27, 2023.

### Study Population

Episodes from individuals were included if they met the following criteria: (1) individuals for whom the AI-based symptom checker showed any of the following 9 potentially related conditions in the first survey data: HIV infection (HIV infection or secondary immunodeficiency disorder) or an STI (trichomoniasis, Chlamydia infection, genital herpes, condyloma acuminatum, gonorrhea, amoebiasis, or syphilis); (2) completion of the revisit survey after the first survey. Individuals were excluded if they answered in the first survey that they were searching for symptoms in someone other than themselves or had been previously diagnosed with HIV infection or secondary immunodeficiency disorder. For individuals with multiple first survey data points during the study period, each data point meeting the above criteria was included to assess the background of the individuals immediately before their medical consultation and testing behavior.

### Outcome Variables

The primary outcome was receiving a medical consultation after answering the AI-based symptom checker, as obtained from the revisit survey data. The secondary outcome was HIV testing among individuals who visited medical care facilities after answering the AI-based symptom checker, which was also obtained from the revisit survey data. A positive HIV test result was obtained from the revisit survey data. Reasons for not undergoing HIV testing and future intentions to undergo testing were evaluated based on HIV-specific banner questionnaire responses.

### Characteristics

To explore the factors associated with the primary and secondary outcomes, several characteristic variables were assessed from the first and revisit survey data. For the first survey data, the variables were age, sex, main symptoms, pain score, STI comorbidities or medical histories, displayed suggested condition, device used to access the website (PC or mobile device), access to HIV awareness information that was displayed during the study period in connection with 9 previously mentioned potential conditions, and survey period. For the revisit survey data, the variables included diagnoses in medical facilities (HIV risk diagnosis in medical facilities, area of medical facilities, facility where the test was conducted, department visited, and place where the HIV test was conducted).

### Statistical Analysis

In this study, the AI-based symptom checker adapted subsequent questions based on individual responses. Therefore, certain questions were presented to only some individuals, resulting in missing data for others. In such cases, a “Do not be asked” category was assigned, and imputation or other missing data handling techniques were not applied.

A flow diagram showing the number of individuals at each stage of the selection process was created. Patient characteristics were summarized according to visit status and HIV testing status. Continuous variables were summarized using means and SDs, or medians and interquartile ranges. Tables with frequencies and percentages were generated for categorical variables. Stratified subgroup analyses were performed.

Multivariate logistic regression analysis was conducted to explore the factors associated with medical consultations. The crude and adjusted odds ratios (ORs) and 95% CIs were estimated. A forest plot of the ORs was also shown. This analysis was conducted in 2 groups: the overall HIV/AIDS/STI–related suggested condition group (suggested HIV/AIDS/STI group) and the STI-related suggested condition subgroup (suggested STI group). The suggested STI group comprised individuals whose symptoms were associated with STI-related suggested conditions. Because HIV/AIDS-related symptom patterns may occur simultaneously in the same individuals who present with STI-related symptoms, the suggested STI group could include individuals who also exhibited HIV/AIDS-related symptom patterns. In this analysis, the suggested STI group was, therefore, treated as a subgroup within the broader suggested HIV/AIDS/STI group. Similarly, factors associated with HIV testing among individuals who received medical consultations were explored using multivariate logistic regression analysis.

To explore HIV-specific information among those who responded to the HIV-specific banner questionnaire, summary statistics of the reasons for not having an HIV test and future intentions to undergo testing were calculated.

All statistical analyses were conducted using Python (version 3.11; Python Software Foundation) and the *statsmodels* library (version 0.14.4).

### Ethical Considerations

This study was approved by the ethics review committee of the Japan Physicians Association (approval 052-2503-02; approved on May 13, 2025).

## Results

**Figure 1.**
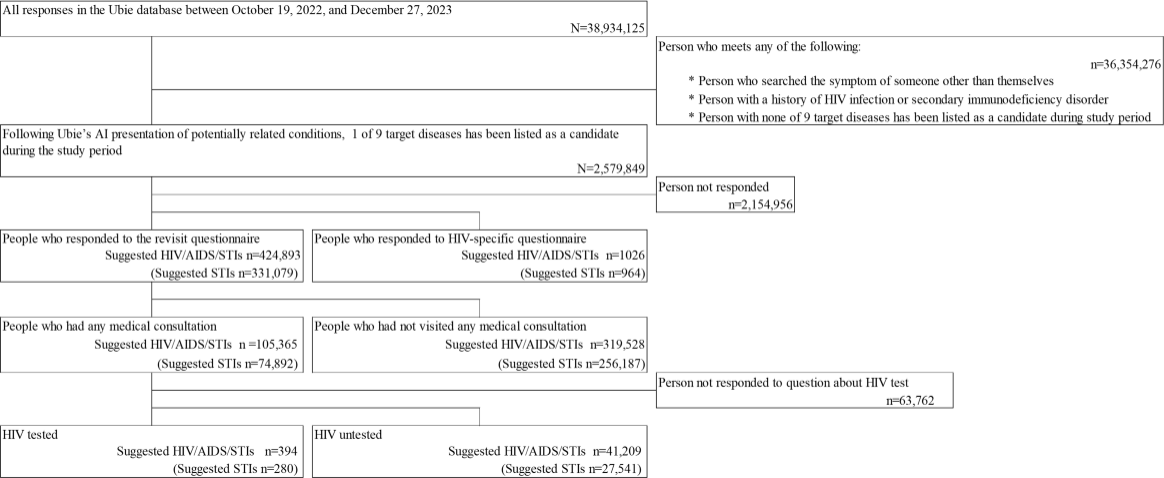
Flow diagram. AI: artificial intelligence; STI: sexually transmitted infection.

The database included 38,934,125 episodes based on the data available from October 19, 2022, to December 27, 2023. Of these, 424,893 eligible episodes from 332,976 individuals (Multimedia Appendix 1) were analyzed (Figure 1). Among these episodes, medical consultations and HIV testing were performed in 105,365 and 394 cases, respectively. In the subset of episodes with suggested STIs, medical consultations and HIV testing were performed in 74,892 and 280 cases, respectively (Figure 1). Patient characteristics for both episodes and individuals are summarized in Table S1 in Multimedia Appendix 1 and were similar across all episodes and individuals.

For the suggested HIV/AIDS/STI group, the median age was 37 (IQR 27-48) years in the group that had a medical consultation and 31 (IQR 22-44) years in the group that did not (Table 1). For the suggested STI group, the median age was 32 (IQR 25-43) years for those who had a medical consultation and 28 (IQR 21-39) years for those who did not. Across all groups, the age category with the highest proportion of episodes was the 20s. Across all groups, 76.2% (80,315/105,365) to 78.5% (201,040/256,187) of the episodes were reported by female users. Genital symptoms were the most frequent main symptoms, reported in 35.8% (37,767/105,365) to 50.4% (37,733/74,892) of episodes. Pain scores were reported as categories “1 to 3” in 10.2% (7605/74,892) to 18.4% (58,696/319,528) of episodes, as categories “4 to 6” in 10.1% (10,613/105,365) to 11.8% (30,196/256,187) of episodes, and as categories “7 to 10” in 3.4 % (10,920/319,528) to 4.9% (3703/74,892) of episodes. The remaining episodes were not assessed for pain because the symptom checker did not determine whether it was necessary to evaluate a possible condition. Over 90% (99,228/105,365; 301,664/319,528; 71,128/74,892; 243,973/256,187) of episodes were accessed via mobile phones. Access to the information page containing HIV educational material was observed in 19.3% (20,344/105,365) to 22.9% (17,127/74892) of the episodes. However, access to the HIV testing information page via the link provided in the educational material was observed in only 0.1% (312/256,187) to 0.2% (175/105,365) of the episodes. The proportion of survey periods was similar across groups.

**Table 1.** Patient characteristics of the suggested HIV/AIDS/STI group and the suggested STI group.

	Suggested HIV/AIDS/STI^a^ group		Suggested STI group^b^	
	Had a medical consultation (n=105,365)	Did not have a medical consultation (n=319,528)	Had a medical consultation (n=74,892)	Did not have a medical consultation (n=256,187)
Age (y), median (IQR)	37 (27-48)	31 (22-44)	32 (25-43)	28 (21-39)
Age group (y), n (%)				
0-19	6617 (6.3)	46,538 (14.6)	5981 (8.0)	44,447 (17.3)
20-29	28,080 (26.7)	100,091 (31.3)	25,926 (34.6)	95,526 (37.3)
30-39	23,302 (22.1)	64,007 (20.0)	18,382 (24.5)	53,958 (21.1)
40-49	23,281 (22.1)	58,164 (18.2)	14,700 (19.6)	39,746 (15.5)
50-59	16,024 (15.2)	34,992 (11.0)	7550 (10.1)	17,967 (7.0)
≥60	8061 (7.7)	15,736 (4.9)	2353 (3.1)	4543 (1.8)
Sex, n (%)				
Male	25,036 (23.8)	72,533 (22.7)	17,006 (22.7)	55,133 (21.5)
Female	80,315 (76.2)	246,970 (77.3)	57,875 (77.3)	201,040 (78.5)
Main symptoms, n (%)^c^				
Genital symptoms	37,767 (35.8)	124,830 (39.1)	37,733 (50.4)	124,778 (48.7)
Symptoms in the ears, nose, throat, eyes, and oral cavity	18,128 (17.2)	49,912 (15.6)	5472 (7.3%)	21,188 (8.3)
Skin and subcutaneous symptoms	10,564 (10.0)	29,485 (9.2)	8089 (10.8)	24,087 (9.4)
Urination-related symptoms	8271 (7.8)	22,960 (7.2)	8271 (11.0)	22,960 (9.0)
General symptoms	7910 (7.5)	14,972 (4.7)	534 (0.7)	1549 (0.6)
Musculoskeletal and joint symptoms	7573 (7.2)	30,543 (9.6)	6259 (8.4)	26,601 (10.4)
Abdominal digestive-related symptoms	7013 (6.7)	26,022 (8.1)	6160 (8.2)	23,785 (9.3)
Pain score, n (%)				
1‐3	19,334 (8.3)	58,696 (18.4)	7605 (10.2)	34,134 (13.3)
4‐6	10,613 (10.1)	37,146 (11.6)	7583 (10.1)	30,196 (11.8)
7‐10	4663 (4.4)	10,920 (3.4)	3703 (4.9)	9097 (3.6)
Not asked	70,755 (67.2)	212,766 (66.6)	56,001 (74.8)	182,760 (71.3)
Device used to access the website (PC or mobile), n (%)				
Mobile	99,228 (94.2)	301,664 (94.4)	71,128 (95.0)	243,973 (95.2)
PC	6133 (5.8)	17,852 (5.6)	3760 (5.0)	12,202 (4.8)
Access to HIV awareness information				
Not open	84,846 (80.5)	256,439 (80.3)	57,641 (77.0)	199,814 (78.0)
Access to the information page on possible diagnoses	20,344 (19.3)	62,694 (19.6)	17,127 (22.9)	56,061 (21.9)
Access to the information page on HIV testing	175 (0.2)	395 (0.1)	124 (0.2)	312 (0.1)
Survey period				
October 19, 2022, to December 31, 2022	16,794 (15.9)	57,568 (18.0)	16,731 (22.3)	57,472 (22.4)
January 1, 2023, to June 30, 2023	45,496 (43.2)	134,301 (42.0)	31,924 (42.6)	106,793 (41.7)
July 1, 2023, to December 27, 2023	43,075 (40.9)	127,659 (40.0)	26,237 (35.0)	91,922 (35.9)

a STI: sexually transmitted infection.

b Population with suggested STIs as possible conditions.

c Symptoms experienced by >5% of visitors were listed.

Approximately 7% (3044/41,603) of episodes in the suggested HIV/AIDS/STI group and 10% (3037/27,821) of episodes in the suggested STI group were diagnosed as actual STIs (Table S2 in Multimedia Appendix 1). Individuals who underwent HIV testing were more likely to have a history of STIs or comorbidities associated with them. Overall, the most frequently visited departments were internal medicine, gynecology, and urology. Among those who underwent HIV testing, more than 50% (373/674) were tested at a hospital. Access to HIV-awareness information ranged from 19.8% (8156/41,209) to 33.6% (94/280) across all groups.

In the suggested STI group, the proportion of men in their 20s was the lowest compared with the other subgroups (Table S3 in Multimedia Appendix 1). Men who have sex with men and individuals with a history of STIs showed a higher proportion of genital symptoms than the general male and female groups. The prevalence of these symptoms among men who have sex with men was 71.7% (370/516) for those who had a medical consultation and 73.3% (1211/1653) for those who did not. The prevalence of these symptoms was 80.7% (5911/7327) and 80.8% (16,438/20,351), respectively, in individuals with a history of STIs. In contrast, the prevalence in the general male and female groups was 35.2% (5978/17,006) and 33.0% (18,177/55,133) and 54.9% (31,749/57,875) and 53.0% (106,596/201,040), respectively. Similarly, the proportion of individuals with urinary symptoms was higher in the high-risk subgroups (men who have sex with men and individuals with a history of STIs). Additionally, access to HIV awareness information was higher in these subgroups than in the general male and female subgroups. Among the suggested STI groups, 10.9% (3037/27821) were actually diagnosed with an STI. In this group, 9.2% (280/3037) underwent an HIV test, and among those tested, 4.6% (13/280) tested positive (Table S4 in Multimedia Appendix 1).

**Figure 2.**
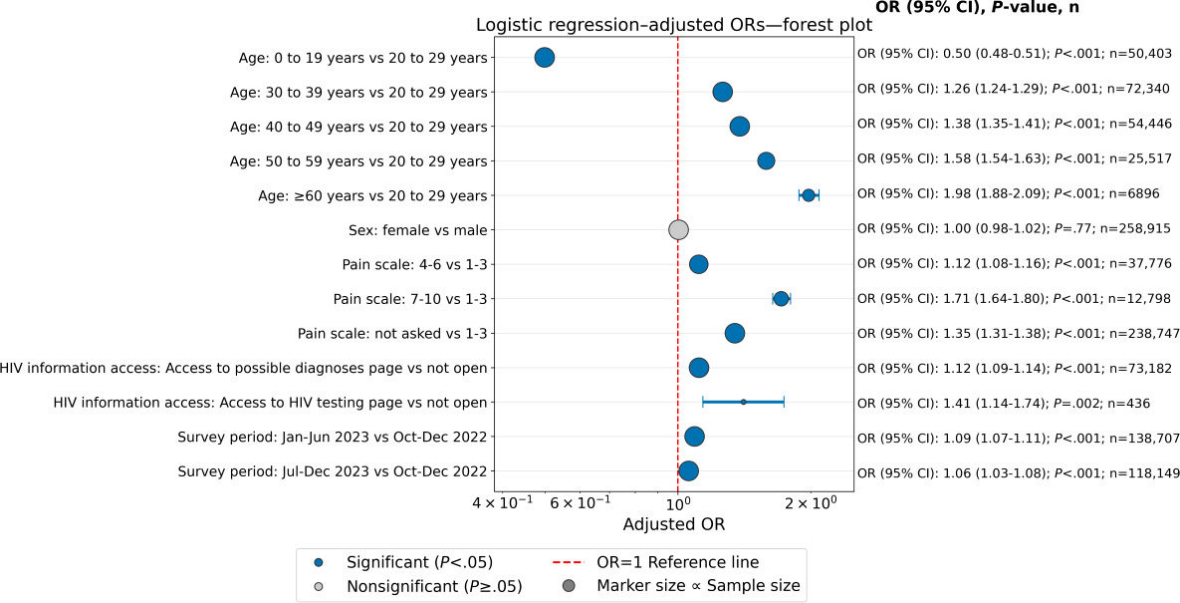
Factors associated with medical consultation (suggested STI group). Forest plot illustrates adjusted odds ratios (ORs) and 95% CIs for medical consultations associated with various factors. The vertical dashed line (OR=1.0) represents the reference line. Blue circles represent *P*<.05, and gray circles represent *P*≥.05. The size of the marker is proportional to the sample size for each category. STI: sexually transmitted infection.

**Figure 3.**
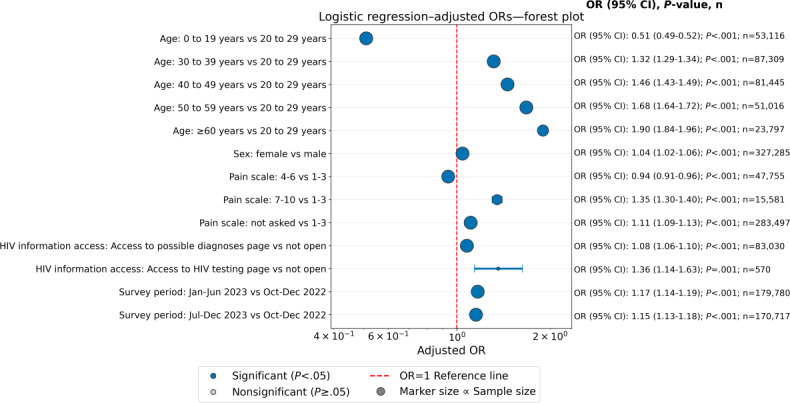
Factors associated with medical consultation (suggested HIV/AIDS/STI group). Forest plot illustrates the adjusted odds ratios (ORs) and 95% CIs for medical consultations associated with various factors. The vertical dashed line (OR=1.0) represents the reference line. Blue circles represent a *P*<.05, and gray circles represent a *P*≥.05. The size of the marker is proportional to the sample size for each category. STI: sexually transmitted infection.

In the suggested STI group, compared with individuals in their 20s, older age groups were significantly more likely to seek medical consultation, with ORs of 1.26 (95% CI 1.24‐1.29) for those aged 30 to 39 years, 1.38 (95% CI 1.35‐1.41) for those aged 40 to 49 years, 1.58 (95% CI 1.54‐1.63) for those aged 50 to 59 years, and 1.98 (95% CI 1.88‐2.09) for those aged 60 years and above (Figure 2). Compared with the low pain score categories (1-3), the higher pain score categories (7-10) were more strongly associated with medical consultation, with an OR of 1.71 (95% CI 1.64‐1.80). Accessing the information page on possible diagnoses, which included HIV awareness content, was positively associated with health care–seeking behaviors, with an OR of 1.12 (95% CI 1.09‐1.14). Accessing the HIV testing page via the link in the HIV awareness section also showed a significant association, with an OR of 1.41 (95% CI 1.14‐1.74). Compared with the 2022 survey period, the 2023 survey period was positively associated with seeking medical consultation, with ORs of 1.09 (95% CI 1.07‐1.11) for January 2023 to June 2023 and 1.06 (95% CI 1.03‐1.08) for July 2023 to December 2023. Similar results were observed in the HIV/AIDS/STI group (Figure 3). In this group, females were more likely to seek medical consultation than males, with an OR of 1.04 (95% CI 1.02‐1.06).

The forest plot illustrates adjusted ORs and 95% CIs for medical consultations associated with various factors. The vertical dashed line (OR=1.0) represents the reference line. Blue circles represent *P*<.05, and gray circles represent *P*≥.05. The size of the marker is proportional to the sample size for each category.

The forest plot illustrates the adjusted ORs and 95% CIs for medical consultations associated with various factors. The vertical dashed line (OR=1.0) represents the reference line. Blue circles represent a *P*<.05, and gray circles represent a *P*≥.05. The size of the marker is proportional to the sample size for each category.

**Figure 4.**
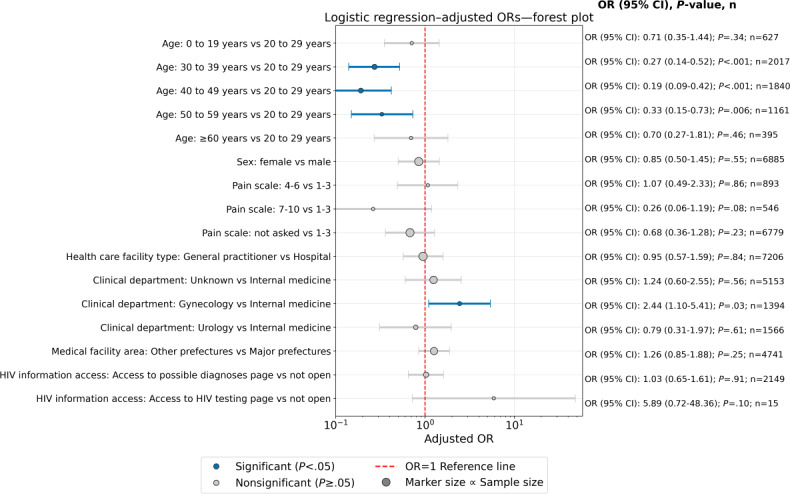
Factors associated with HIV testing (suggested STI group). The forest plot illustrates the adjusted odds ratios (ORs) and 95% CIs for HIV testing associated with various factors. The vertical dashed line (OR=1.0) represents the reference line. Blue circles represent *P*<.05, and gray circles represent *P*≥.05. The size of the marker is proportional to the sample size for each category. STI: sexually transmitted infection.

**Figure 5.**
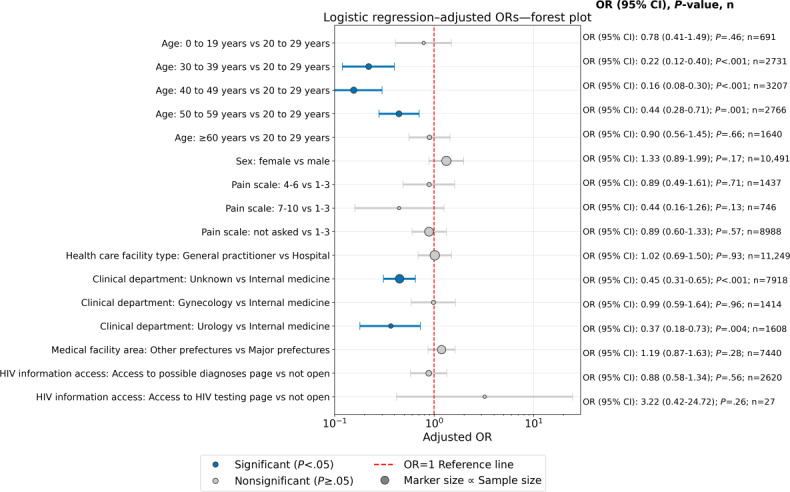
Factors associated with HIV testing (suggested HIV/AIDS/STI group). The forest plot illustrates the adjusted odds ratios (ORs) and 95% CIs for HIV testing associated with various factors. The vertical dashed line (OR=1.0) represents the reference line. Blue circles represent *P*<.05, and gray circles represent *P*≥.05. The size of the marker is proportional to the sample size for each category. STI: sexually transmitted infection.

In contrast to the results of medical consultation behavior, the 30 to 59 age category was negatively associated with HIV testing behavior compared with the 20‐29 age category in the suggested STI group, with ORs of 0.27 (95% CI 0.14‐0.52) for those aged 30 to 39 years, 0.19 (95% CI 0.09‐0.42) for those aged 40 to 49 years, and 0.33 (95% CI 0.15‐0.73) for those aged 50 to 59 years (Figure 4). No significant associations were observed between HIV testing and factors, such as sex, pain score, or exposure to awareness information. Similarly, neither the type of facility visited nor the geographical area of the medical facility was associated with HIV-testing behavior. Compared with internal medicine departments, gynecology departments were significantly associated with HIV testing, with an OR of 2.44 (95% CI 1.10‐5.41). In the suggested HIV/AIDS/STI group, the urology department was negatively associated with HIV testing, with an OR of 0.37 (95% CI 0.18‐0.73; Figure 5).

The forest plot illustrates the adjusted ORs and 95% CIs for HIV testing associated with various factors. The vertical dashed line (OR=1.0) represents the reference line. Blue circles represent *P*<.05, and gray circles represent *P*≥.05. The size of the marker is proportional to the sample size for each category.

The forest plot illustrates the adjusted ORs and 95% CIs for HIV testing associated with various factors. The vertical dashed line (OR=1.0) represents the reference line. Blue circles represent *P*<.05, and gray circles represent *P*≥.05. The size of the marker is proportional to the sample size for each category.

The most frequent reason selected for not undergoing an HIV test was “I did not feel it was necessary,” with a proportion of 35.3% (54/153) to 49.6% (59/119) across the subgroups. High-risk populations with a history of STIs showed a lower proportion of this response than other subgroups (Table 2). Additionally, 11.3% (109/964) of the respondents did not know where to get tested, and 4.9% (47/964) were unaware of the availability of HIV testing. The proportion of those who felt barriers to receiving an HIV test was 4.2% (35/838) for females and 0.8% (1/119) for males. Concerns about the cost of HIV testing were reported by 5.4% (52/964) of respondents.

**Table 2.** Reasons for not undergoing an HIV test among the suggested STI^a^ group.

	All (n=964), n (%)	Male (n=119), n (%)	Female (n=838), n (%)	STI^a^ history (positive; n=153), n (%)
I did not feel it was necessary	437 (45.3)	59 (49.6)	374 (44.6)	54 (35.3)
I did not have time to get an HIV test	81 (8.4)	14 (11.8)	66 (7.9)	14 (9.2)
I did not know where to get an HIV test	109 (11.3)	9 (7.6)	100 (11.9)	22 (14.4)
I did not know an HIV test was available	47 (4.9)	5 (4.2)	42 (5.0)	8 (5.2)
I felt there were barriers to getting an HIV test	36 (3.7)	1 (0.8)	35 (4.2)	4 (2.6)
I thought it would be expensive to get an HIV test	52 (5.4)	5 (4.2)	47 (5.6)	11 (7.2)
Others	254 (26.3)	31 (26.1)	221 (26.4)	51 (33.3)

a STI: sexually transmitted infection.

The intention to undergo an HIV test was positively influenced in 71.0% (685/964) of the individuals, and 29.0% (280/964) of those who originally had no intention of being tested changed their minds and decided to undergo an HIV test (Table 3). The male population had the highest proportion of individuals who had never been tested for HIV infection (43/119, 36.1%).

**Table 3.** Intention to undergo HIV testing after an AI^a^-based symptom check in the suggested STI^b^ group.

	All (n=964), n (%)	Male (n=119), n (%)	Female (n=838), n (%)	STI^a^ history (positive; n=153), n (%)
Did you change your mind about the HIV test after AI^a^ algorithm–based information provision?				
Never intended to get an HIV test	259 (26.9)	43 (36.1)	212 (25.3)	37 (24.2)
Initially no intention, but now I understand the importance, but I will not undergo an HIV test	236 (24.5)	22 (18.5)	213 (25.4)	39 (25.5)
Initially no intention, but now I will undergo an HIV test	280 (29.0)	31 (26.1)	248 (29.6)	42 (27.5)
Intention to undergo an HIV test has increased	169 (17.5)	22 (18.5)	146 (17.4)	35 (22.9)
Others	20 (2.1)	1 (0.8)	19 (2.3)	0 (0.0)

a AI: artificial intelligence.

b STI: sexually transmitted infection.

A total of 20.8% (200/964) of the participants were aware of HIV postal testing; however, only 1.6% (15/964) had experience in using it (Table S5 in Multimedia Appendix 1). A total of 18.7% (180/964) of individuals answered that they did not intend to receive a test but would be willing to use HIV postal testing. The distribution of answers was generally similar across male, female, and high-risk populations (those with a history of STIs). The men who have sex with men subgroup was excluded from the analysis due to the small number of patients (n=8).

## Discussion

### Principal Findings

Our study analyzed factors associated with subsequent medical consultation and HIV testing among users who were identified by an AI-based symptom checker as having symptoms potentially related to HIV risk. We found that older age, higher pain severity, and access to HIV-related awareness information were associated with a greater likelihood of seeking medical consultation. Although HIV testing was more frequently performed in gynecology departments than in internal medicine, testing rates remained low across all major clinical departments, particularly in urology. Use of the AI-based symptom checker was also associated with an improvement in users’ intention to undergo HIV testing. These findings suggest the importance of improving access to and optimizing HIV-related awareness information for at-risk individuals, together with strengthening HIV testing practices within clinical settings.

### Benefit of Approaching This Population

Using data from a widely used AI-based symptom checker in Japan, this study identified key populations that should be prioritized for HIV testing. Among the 420,000 episodes analyzed, approximately 330,000 involved suggested STIs, which are critical for the early detection of HIV infection. Of these, confirmed STI diagnoses were reported in approximately 10% (3037/331,079) of individuals who sought medical care, with a total of approximately 3000 cases. Considering the possibility that a certain number of at-risk individuals existed among those who did not report their consultation outcomes, it is likely that the system effectively reached key populations. In addition, men who have sex with men and individuals with a history of STIs showed a higher prevalence of genital and urinary symptoms than the general male and female subgroups. Furthermore, the proportion of access to awareness materials was higher among high-risk populations. These findings suggest that within these high-risk populations, individuals in need of HIV testing may have been more accurately identified than those in other subgroups. Previous studies have reported that being a man who has sex with men is associated with an early HIV diagnosis [8,9]. Higher engagement with awareness materials among these groups may reflect the greater relevance of the content to their specific risks.

This study is also noteworthy for successfully addressing a previously underexplored demographic gap and providing valuable insights. First, it includes users from rural areas, including those outside government-designated cities, at levels comparable to or even exceeding those from urban regions. Previous reports have suggested that promoting HIV testing and improving diagnostic rates in rural areas are key public health challenges in Japan [8], as diagnosis rates tend to be higher in urban areas and lower in rural regions. This suggests that this study may have successfully reached populations with a greater need for awareness and promotion of HIV testing. Second, this study included a high proportion of females in the high-risk population. However, in Japan, women are a relatively overlooked group in HIV diagnosis, accounting for only 3.5% (19/539) of newly diagnosed HIV cases in recent years [4]. Consequently, there is a prevailing perception that HIV is not relevant to women, and awareness campaigns tend to exclude them. This study successfully identified a previously underrepresented population as well as possible HIV-positive cases.

### Factors Associated With Medical Consultation and HIV Testing (Except Awareness Activity)

This study provides insights into several factors associated with medical consultations and HIV testing behaviors. Individuals in their 20s showed a lower tendency to seek medical consultations than older age groups. A previous study investigating factors related to late HIV diagnosis reported that older age is associated with late diagnosis [8], which contrasts with the findings of our study. This difference is likely because these studies evaluated late diagnoses based on CD4 counts at the time of HIV diagnosis, whereas our study assessed behavior changes following recommendations from an AI-based symptom checker. In contrast to medical consultation behavior, a higher proportion of individuals in their 20s underwent HIV testing after medical consultation compared with older age groups. This suggests that, compared with other age groups, individuals in their 20s are more likely to undergo HIV testing once they seek medical care. Given that new HIV infections are most frequently reported among individuals in their 30s, followed by those in their 20s, promoting health care–seeking behaviors among people in their 20s is particularly important [4]. Strengthening HIV screening for individuals in their 20s who present with the suggested STIs is crucial, and the key challenge lies in effectively encouraging this population to seek medical attention. For cases with suggested STIs, HIV testing was more frequent in the gynecology department than in the internal medicine department, whereas no significant differences were observed between the urology and internal medicine departments. This may be because HIV screening is routinely conducted in all pregnant women in Japan, which could contribute to a lower barrier to testing in gynecology departments. However, the testing rates among the suggested STI groups were low across all specialties, remaining at 0.92% (9/975) in internal medicine, 1.75% (54/3090) in gynecology, and even lower in urology (0.19%, 3/1576). In Japan, several practical barriers may contribute to low HIV testing in STI-related clinics, including urology. First, physicians often find it burdensome to explain and recommend HIV testing to patients who do not request it themselves. Second, because free and anonymous HIV testing is available at public health centers, which also handle posttest procedures, clinicians have limited incentive to perform testing within their own facilities. Third, awareness of insurance coverage for HIV testing in STI-related contexts remains low, and concerns about reimbursement or claim denial persist [15]. In addition, in urology practice, genital symptoms often lead to a relatively clear presumptive diagnosis and prompt treatment of specific STIs, which may result in clinical focus being placed on immediate disease management rather than on broader risk assessment, including HIV testing. This highlights the need for renewed awareness among urology health care providers that the presence of STIs should also prompt consideration of concurrent HIV testing. Barriers to HIV testing need to be addressed and improved.

This study provides a detailed investigation of the barriers to HIV testing among a subset of users who responded to an HIV-specific banner questionnaire. One of the most common reasons for not undergoing HIV testing was the belief that HIV was not relevant to oneself, which was reported by approximately 40% (54/153) to 50% (59/119) of the respondents. In addition, 11.3% (109/964) of the respondents did not know where to get tested, and 4.9% (47/964) were unaware that HIV testing existed. Lack of time and concern about cost were cited as reasons for not being tested by 8.4% (81/964) and 5.4% (52/964) of the respondents, respectively. Moreover, 3.7% (36/964) reported feeling that there were barriers to undergoing HIV testing. These findings suggest that a lack of knowledge regarding HIV risk and testing options may contribute to the absence of testing behavior. These results are consistent with those of previous studies conducted among men who have sex with men populations, reinforcing the importance of recognizing one’s own risk of HIV infection and gaining knowledge about HIV testing [16]. This study also found that 20.8% (200/964) of the respondents were aware of the availability of mail-based HIV testing services. This suggests that promoting awareness of various testing options may help encourage testing behavior, particularly in populations with low awareness.

### Benefit of Education Through the Symptom Checker

This study demonstrated the benefits and challenges of information provision approaches related to HIV testing. The population in this study consisted of individuals who exhibited certain symptoms and were therefore more likely to visit medical institutions or testing facilities than asymptomatic individuals. Approximately 25% (105,365/424,893) of the participants visited a medical institution. In the study population, the provision of information through an AI-based symptom checker was identified as a factor associated with health care–seeking behaviors. Additionally, among respondents to the HIV-specific banner questionnaire, approximately 29% (280/964) reported a change in mindset; that is, participants became more receptive to HIV testing despite not having initially intended to undergo it. Furthermore, 18.7% (180/964) expressed a new intention to use mail-based HIV-testing services. Based on these findings, when educational information reaches users, it has the potential to promote medical consultations and testing behaviors to a certain extent, thereby demonstrating the usefulness of web-based awareness initiatives. However, these findings indicate that there is room for improvement in information accessibility, as only approximately 20% (156,226/755,972) of users viewed HIV-awareness content related to their HIV-related conditions, and only approximately 0.1% (1006/755,972) accessed pages about HIV testing. Therefore, strategies to increase visibility and engagement with educational content are needed.

While the provision of information through AI-based symptom checkers was associated with health care–seeking behaviors, no significant association was found with HIV testing uptake in this study. A previous survey conducted at 11 clinics in Japan that handle STIs revealed that 6 did not offer HIV testing unless requested by the patient [15]. These findings suggest that there are barriers to HIV testing in medical institutions, such as the need to explain and persuade patients who do not proactively request testing and the lack of incentives. In the United States, a previous study on the medical records of newly diagnosed HIV cases reported that missed opportunities for HIV testing were common. Therefore, strengthening opt-out HIV screening programs is recommended [17]. These findings, along with ours, suggest that health care providers should adopt a proactive approach to information provision. Individuals must also be encouraged to recognize their potential risks and take the initiative to request testing or select their preferred testing method. Our results highlight the importance of raising awareness among physicians and patients to reduce barriers to HIV testing in the current Japanese setting.

### Limitations

This study has some limitations. First, because the number of HIV testing events was very small (394/424,893 cases, 0.09%), the stability and precision of the regression estimates are limited. Although a commonly cited rule suggests that approximately 10 events per variable may be acceptable for multivariable logistic regression, the rarity of the outcome in this study raises concerns regarding estimate stability. Therefore, the regression analyses were conducted for exploratory purposes to identify general patterns of association rather than to provide precise effect estimates, and the results should be interpreted with caution.

Second, this study used an episode-based analytic approach, which may introduce within-person dependency when individuals contributed multiple episodes. Although baseline characteristics were similar when summarized at the individual and episode levels, the potential nonindependence across episodes may affect the estimation of variance for the estimates, representing a limitation of this study.

Third, the data source for this study did not allow for the precise identification of individuals with a history of HIV infection at the time of the first survey. Therefore, it was difficult to completely exclude all individuals with a prior HIV diagnosis from the study population, and some may have been included. Consequently, there are limitations in generalizing the study population to the intended target population, which consists of individuals with possible HIV infection or STIs who have not previously received medical consultation or testing. However, before the 9 relevant, potentially related conditions were displayed in the first survey, data on whether individuals had been diagnosed with each condition were obtained. This allowed for the exclusion of individuals with HIV infection or STIs.

Fourth, outcome evaluation relied on revisit survey data; therefore, only individuals who responded to the survey were included. This may have introduced a selection bias in the study population. For instance, individuals who received medical consultation after the first survey but did not revisit the AI-based symptom checker were excluded, potentially leading to an underestimation of medical consultations. Additionally, if individuals received medical consultation after revisiting the AI-based symptom checker, they may still be misclassified as not having received medical consultation in the revisit survey data, further contributing to an underestimation of medical consultations. However, this system prevents revisit surveys within 24 hours of the first survey, which may mitigate bias by excluding immediate revisits. Moreover, because approximately 80% to 90% of individuals did not complete the revisit survey and were therefore excluded from the analysis, it is difficult to generalize the findings to this large excluded population, reflecting potential selection and attrition bias.

Fifth, the first survey data in this study were derived from an AI-based symptom checker questionnaire that adapted subsequent questions based on individual responses. Consequently, certain questions, such as those regarding comorbidities or symptoms, were presented only to specific individuals, leading to missing data. Missing data reflect the absence of questioning rather than the absence of a condition or symptom, which limits the assessment of factors associated with the outcomes. Furthermore, although transgender individuals were considered a high-risk population, they were not identified in this study. Consequently, some women may have been categorized as female. A further limitation is that the internal algorithms, versioning, and update processes of the AI-based symptom checker are proprietary and were not fully available for independent reporting in this study. As a result, we were unable to detail the specific AI methods or evaluate how algorithmic updates or postdeployment monitoring might influence system behavior. Our description of the tool is, therefore, limited to its observable adaptive question flow behavior, and the findings should be interpreted with this constraint in mind.

Sixth, as this data source was derived from a self-reported questionnaire, the outcomes and characteristic variables obtained may be subject to misclassification owing to individual recall errors. As the results of HIV testing are based on self-reporting from revisit surveys, it is unclear whether they reported the results of the screening test or a definitive diagnosis. This could have caused an overestimation of HIV-positive cases. This overestimation is more likely to occur in populations with a low underlying HIV prevalence, such as women and heterosexual men in the Japanese epidemiological context, where false-positive screening results are more common relative to confirmed HIV diagnoses.

### Conclusions

This study suggests that HIV-related information provided through an AI-based symptom checker may influence individuals’ awareness or consideration of medical consultation. To further promote HIV testing, it may be necessary to refine the content and delivery of educational materials and enhance HIV testing literacy among physicians who encounter patients with STIs. In addition, such tools may be useful when integrated into broader HIV awareness or testing promotion strategies led by community-based organizations or the public sector.

## Supplementary material

10.2196/90257 Multimedia Appendix 1 Supplementary tables and figures supporting the main analyses and the plain language summary.

## Abbreviations

AI: artificial intelligence
OR: odds ratio
STI: sexually transmitted infection
