# Supplementary material for: Medical Consultation and HIV Testing After AI-Based Symptom Check: Retrospective Cohort Study
Source: J Med Internet Res. 2026 Jun 18;28:e90257. doi: 10.2196/90257 (PMC13278609; doi:10.2196/90257)

Multimedia Appendix 1

Table S1. Characteristics of episodes and individuals

|  |  | Episodes | |  | Individuals | |
| --- | --- | --- | --- | --- | --- | --- |
|  |  | n = | 424,893 |  | n = | 332,976 |
| Age | |  |  |  |  |  |
|  | mean (SD) | 35.15 | 14.2 |  | 35.81 | 14.3 |
|  | median (1Q,3Q) | 33 | 23, 45 |  | 34 | 24, 46 |
| Age group, n (%) | |  |  |  |  |  |
|  | 0 to 19 years | 53,155 | 12.5% |  | 39,648 | 11.9% |
|  | 20 to 29 years | 128,171 | 30.2% |  | 93,794 | 28.2% |
|  | 30 to 39 years | 87,309 | 20.5% |  | 70,379 | 21.1% |
|  | 40 to 49 years | 81,445 | 19.2% |  | 67,392 | 20.2% |
|  | 50 to 59 years | 51,016 | 12.0% |  | 42,077 | 12.6% |
|  | >=60 years | 23,797 | 5.6% |  | 19,686 | 5.9% |
| Sex, n (%) | |  |  |  |  |  |
|  | Male | 97,569 | 23.0% |  | 77,794 | 23.4% |
|  | Female | 327,285 | 77.0% |  | 255,150 | 76.6% |
|  | Unknown | 39 | 0.0% |  | 32 | 0.0% |
| Main symptoms, n (%) | |  |  |  |  |  |
|  | Genital symptoms | 162,597 | 38.3% |  | 115,995 | 34.8% |
|  | Symptoms in the ears, nose, throat, eyes, and oral cavity | 68,040 | 16.0% |  | 58,009 | 17.4% |
|  | Skin and subcutaneous symptoms | 40,049 | 9.4% |  | 31,615 | 9.5% |
| Pain score, n (%) | |  |  |  |  |  |
|  | 1-3 | 78,030 | 18.4% |  | 64,334 | 19.3% |
|  | 4-6 | 47,759 | 11.2% |  | 38,980 | 11.7% |
|  | 7-10 | 15,583 | 3.7% |  | 12,981 | 3.9% |
|  | Not asked | 283,521 | 66.7% |  | 216,681 | 65.1% |
| STI Comorbidities/ Medical histories, n (%) | |  |  |  |  |  |
|  | Sexually transmitted infections (STIs) | 20,003 | 4.7% |  | 13,751 | 4.1% |
| Displayed possible condition (HIV-related condition), n (%) | |  |  |  |  |  |
|  | Acute HIV infection | 2,259 | 0.5% |  | 1,939 | 0.6% |
|  | Secondary immunodeficiency syndrome | 93,503 | 22.0% |  | 80,734 | 24.2% |
|  | Trichomoniasis | 122,648 | 28.9% |  | 89,709 | 26.9% |
|  | Chlamydia infection | 177,296 | 41.7% |  | 133,260 | 40.0% |
|  | Genital herpes | 70,326 | 16.6% |  | 53,021 | 15.9% |
|  | Condyloma acuminatum | 27,421 | 6.5% |  | 19,860 | 6.0% |
|  | Gonorrhea | 30,942 | 7.3% |  | 22,785 | 6.8% |
|  | Amoebiasis | 1,142 | 0.3% |  | 985 | 0.3% |
|  | Syphilis | 21,011 | 4.9% |  | 16,339 | 4.9% |
| Displayed possible condition (Others), n (%) | |  |  |  |  |  |
|  | Uterine prolapse | 100,149 | 23.6% |  | 53,565 | 16.1% |
|  | Genital eczema | 71,607 | 16.9% |  | 51,663 | 15.5% |
|  | Acute tonsillitis and pharyngitis | 62,562 | 14.7% |  | 36,624 | 11.0% |
| Device used to access the website (PC / Mobile), n (%) | |  |  |  |  |  |
|  | Mobile | 400,892 | 94.4% |  | 312,333 | 93.8% |
|  | PC | 23,985 | 5.6% |  | 20,635 | 6.2% |
|  | Unknown | 16 | 0.0% |  | 8 | 0.0% |
| Access to HIV awareness information | |  |  |  |  |  |
|  | Not open | 341,285 | 80.3% |  | 273,608 | 82.2% |
|  | Access to the information page on possible diagnoses | 83,038 | 19.5% |  | 58,973 | 17.7% |
|  | Access to the information page on HIV testing | 570 | 0.1% |  | 395 | 0.1% |
| Survey period | |  |  |  |  |  |
|  | October 19, 2022 - December 31, 2022 | 74,362 | 17.5% |  | 54,877 | 16.5% |
|  | January 1, 2023 - June 30, 2023 | 179,797 | 42.3% |  | 137,126 | 41.2% |
|  | July 1, 2023 - December 27, 2023 | 170,734 | 40.2% |  | 140,973 | 42.3% |

Table S2. Characteristics of individuals who visited medical facilities, stratified by HIV testing status

|  |  | Suggested HIV/AIDS/STI group | | | |  | Suggested STI group | | | |
| --- | --- | --- | --- | --- | --- | --- | --- | --- | --- | --- |
|  |  | Did not undergo an HIV test | | Underwent an HIV test | |  | Did not undergo an HIV test | | Underwent an HIV test | |
|  |  | n = | 41,209 | n = | 394 |  | n = | 27,541 | n = | 280 |
| Age | |  |  |  |  |  |  |  |  |  |
|  | mean (SD) | 38 | 13.9 | 35 | 15.8 |  | 34 | 12.2 | 29 | 10.8 |
|  | median (1Q,3Q) | 37 | 27, 48 | 29 | 23, 46 |  | 31 | 24, 43 | 26 | 22, 33 |
| Age group, n (%) | |  |  |  |  |  |  |  |  |  |
|  | 0 to 19 years | 2,345 | 5.7% | 32 | 8.1% |  | 2,065 | 7.5% | 29 | 10.4% |
|  | 20 to 29 years | 11,201 | 27.2% | 166 | 42.1% |  | 10,141 | 36.8% | 156 | 55.7% |
|  | 30 to 39 years | 9,135 | 22.2% | 74 | 18.8% |  | 6,687 | 24.3% | 56 | 20.0% |
|  | 40 to 49 years | 9,239 | 22.4% | 33 | 8.4% |  | 5,230 | 19.0% | 22 | 7.9% |
|  | 50 to 59 years | 6,294 | 15.3% | 45 | 11.4% |  | 2,646 | 9.6% | 11 | 3.9% |
|  | >=60 years | 2,995 | 7.3% | 44 | 11.2% |  | 772 | 2.8% | 6 | 2.1% |
| Sex, n (%) | |  |  |  |  |  |  |  |  |  |
|  | Male | 9,323 | 22.6% | 101 | 25.6% |  | 5,877 | 21.3% | 69 | 24.6% |
|  | Female | 31,880 | 77.4% | 293 | 74.4% |  | 21,661 | 78.7% | 211 | 75.4% |
|  | Unknown | 6 | 0.0% | 0 | 0.0% |  | 3 | 0.0% | 0 | 0.0% |
| Main symptoms, n (%) | |  |  |  |  |  |  |  |  |  |
|  | Genital symptoms | 14,582 | 35.4% | 190 | 48.2% |  | 14,567 | 52.9% | 190 | 67.9% |
|  | Symptoms in the ears, nose, throat, eyes, and oral cavity | 7,710 | 18.7% | 43 | 10.9% |  | 2,015 | 7.3% | 18 | 6.4% |
|  | Urination-related symptoms | 3,810 | 9.2% | 12 | 3.0% |  | 3,810 | 13.8% | 12 | 4.3% |
|  | General symptoms | 3,806 | 9.2% | 53 | 13.5% |  | 191 | 0.7% | 3 | 1.1% |
|  | Skin and subcutaneous symptoms | 3,188 | 7.7% | 25 | 6.3% |  | 2,398 | 8.7% | 19 | 6.8% |
| Pain score, n (%) | |  |  |  |  |  |  |  |  |  |
|  | 1-3 | 8,400 | 20.4% | 84 | 21.3% |  | 2,657 | 9.6% | 29 | 10.4% |
|  | 4-6 | 3,927 | 9.5% | 34 | 8.6% |  | 2,548 | 9.3% | 28 | 10.0% |
|  | 7-10 | 1,787 | 4.3% | 10 | 2.5% |  | 1,388 | 5.0% | 5 | 1.8% |
|  | Not asked | 27,095 | 65.8% | 266 | 67.5% |  | 20,948 | 76.1% | 218 | 77.9% |
| STI Comorbidities/ Medical histories, n (%) | |  |  |  |  |  |  |  |  |  |
|  | Sexually transmitted infections (STIs) | 2,939 | 7.1% | 57 | 14.5% |  | 2,880 | 10.5% | 57 | 20.4% |
| Device used to access the website (PC / Mobile), n (%) | |  |  |  |  |  |  |  |  |  |
|  | Mobile | 38,974 | 94.6% | 371 | 94.2% |  | 26,274 | 95.4% | 263 | 93.9% |
|  | PC | 2,235 | 5.4% | 23 | 5.8% |  | 1,267 | 4.6% | 17 | 6.1% |
| Diagnosis in medical facility (HIV risk diagnosis), n (%) | |  |  |  |  |  |  |  |  |  |
|  | Acute HIV infection | 17 | 0.0% | 5 | 1.3% |  | 11 | 0.0% | 2 | 0.7% |
|  | Secondary immunodeficiency syndrome | 18 | 0.0% | 0 | 0.0% |  | 0 | 0.0% | 0 | 0.0% |
|  | Sexually transmitted infections (STIs) | 3,015 | 7.3% | 29 | 7.4% |  | 3,008 | 10.9% | 29 | 10.4% |
| Area of Medical facilities, n (%) | |  |  |  |  |  |  |  |  |  |
|  | Prefectures with cities designated by government ordinance | 6,860 | 16.6% | 71 | 18.0% |  | 4,297 | 15.6% | 42 | 15.0% |
|  | Other prefectures | 7,349 | 17.8% | 91 | 23.1% |  | 4,681 | 17.0% | 60 | 21.4% |
|  | Unknown | 27,000 | 65.5% | 232 | 58.9% |  | 18,563 | 67.4% | 178 | 63.6% |
| Facility where the test was conducted, n (%) | |  |  |  |  |  |  |  |  |  |
|  | Hospital | 3,088 | 7.5% | 33 | 8.4% |  | 1,855 | 6.7% | 19 | 6.8% |
|  | General Practitioner | 11,121 | 27.0% | 129 | 32.7% |  | 7,123 | 25.9% | 83 | 29.6% |
|  | Unknown | 27,000 | 65.5% | 232 | 58.9% |  | 18,563 | 67.4% | 178 | 63.6% |
| The department visited, n (%) | |  |  |  |  |  |  |  |  |  |
|  | Internal Medicine | 3,397 | 8.2% | 59 | 15.0% |  | 966 | 3.5% | 9 | 3.2% |
|  | Gynecology | 3,084 | 7.5% | 54 | 13.7% |  | 3,036 | 11.0% | 54 | 19.3% |
|  | Urology | 1,615 | 3.9% | 3 | 0.8% |  | 1,573 | 5.7% | 3 | 1.1% |
|  | Other | 4,284 | 10.4% | 22 | 5.6% |  | 1,764 | 6.4% | 11 | 3.9% |
|  | No response | 28,829 | 70.0% | 248 | 62.9% |  | 20,202 | 73.4% | 195 | 69.6% |
| Place where the HIV test was conducted, n (%) | |  |  |  |  |  |  |  |  |  |
|  | Hospital | 0 | 0.0% | 223 | 56.6% |  | 0 | 0.0% | 150 | 53.6% |
|  | Home | 0 | 0.0% | 78 | 19.8% |  | 0 | 0.0% | 44 | 15.7% |
|  | Public health center or testing facility | 0 | 0.0% | 37 | 9.4% |  | 0 | 0.0% | 31 | 11.1% |
|  | Not specified | 0 | 0.0% | 56 | 14.2% |  | 0 | 0.0% | 55 | 19.6% |
|  | No response | 41,209 | 100.0% | 0 | 0.0% |  | 27,541 | 100.0% | 0 | 0.0% |
| Access to HIV awareness information | |  |  |  |  |  |  |  |  |  |
|  | Not open | 32,984 | 80.0% | 283 | 71.8% |  | 20,606 | 74.8% | 185 | 66.1% |
|  | Access to the information page on possible diagnoses | 8,156 | 19.8% | 110 | 27.9% |  | 6,889 | 25.0% | 94 | 33.6% |
|  | Access to the information page on HIV testing | 69 | 0.2% | 1 | 0.3% |  | 46 | 0.2% | 1 | 0.4% |
| Survey period | |  |  |  |  |  |  |  |  |  |
|  | October 19, 2022 - December 31, 2022 | 5,213 | 12.7% | 48 | 12.2% |  | 5,186 | 18.8% | 47 | 16.8% |
|  | January 1, 2023 - June 30, 2023 | 16,543 | 40.1% | 161 | 40.9% |  | 11,133 | 40.4% | 110 | 39.3% |
|  | July 1, 2023 - December 27, 2023 | 19,453 | 47.2% | 185 | 47.0% |  | 11,222 | 40.7% | 123 | 43.9% |

Table S3. Characteristics of the suggested STI group

|  |  |  | Male | | | |  | Female | | | |  | MSM ^a^ | | | |  | STIs ^b^ history (+) | | | |
| --- | --- | --- | --- | --- | --- | --- | --- | --- | --- | --- | --- | --- | --- | --- | --- | --- | --- | --- | --- | --- | --- |
|  |  |  | Had any medical  consultation | | Did not had any medical  consultation | |  | Had any medical  consultation | | Did not had any medical  consultation | |  | Did not had any medical  consultation | | Did not had any medical  consultation | |  | Had any medical  consultation | | Did not had any medical  consultation | |
|  |  |  | n = | 17,006 | n = | 55,133 |  | n = | 57,875 | n = | 201,040 |  | n = | 516 | n = | 1,653 |  | n = | 7,327 | n = | 20,351 |
| Age | | |  |  |  |  |  |  |  |  |  |  |  |  |  |  |  |  |  |  |  |
|  | median (1Q,3Q) |  | 36 | 27, 47 | 32 | 24, 44 |  | 31 | 24, 42 | 27 | 21, 38 |  | 28 | 22, 37 | 25 | 20, 35 |  | 29 | 24, 38 | 27 | 21, 35 |
| Age group, n (%) | | |  |  |  |  |  |  |  |  |  |  |  |  |  |  |  |  |  |  |  |
|  | 0 to 19 years |  | 1,034 | 6.1% | 6,509 | 11.8% |  | 4,936 | 8.5% | 37,924 | 18.9% |  | 59 | 11.4% | 370 | 22.4% |  | 493 | 6.7% | 2,746 | 13.5% |
|  | 20 to 29 years |  | 4,542 | 26.7% | 16,891 | 30.6% |  | 21,384 | 36.9% | 78,635 | 39.1% |  | 216 | 41.9% | 677 | 41.0% |  | 3,307 | 45.1% | 9,823 | 48.3% |
|  | 30 to 39 years |  | 4,341 | 25.5% | 13,180 | 23.9% |  | 14,041 | 24.3% | 40,778 | 20.3% |  | 138 | 26.7% | 314 | 19.0% |  | 1,931 | 26.4% | 4,345 | 21.4% |
|  | 40 to 49 years |  | 3,822 | 22.5% | 10,682 | 19.4% |  | 10,878 | 18.8% | 29,064 | 14.5% |  | 69 | 13.4% | 216 | 13.1% |  | 1,117 | 15.2% | 2,499 | 12.3% |
|  | 50 to 59 years |  | 2,214 | 13.0% | 5,741 | 10.4% |  | 5,336 | 9.2% | 12,226 | 6.1% |  | 25 | 4.8% | 63 | 3.8% |  | 402 | 5.5% | 830 | 4.1% |
|  | >=60 years |  | 1,053 | 6.2% | 2,130 | 3.9% |  | 1,300 | 2.2% | 2,413 | 1.2% |  | 9 | 1.7% | 13 | 0.8% |  | 77 | 1.1% | 108 | 0.5% |
| Sex, n (%) | | |  |  |  |  |  |  |  |  |  |  |  |  |  |  |  |  |  |  |  |
|  | Male |  | 17,006 | 100.0% | 55,133 | 100.0% |  | 0 | 0.0% | 0 | 0.0% |  | 118 | 22.9% | 379 | 22.9% |  | 1,643 | 22.4% | 3,972 | 19.5% |
|  | Female |  | 0 | 0.0% | 0 | 0.0% |  | 57,875 | 100.0% | 201,040 | 100.0% |  | 398 | 77.1% | 1,273 | 77.0% |  | 5,684 | 77.6% | 16,377 | 80.5% |
|  | Unknown |  | 0 | 0.0% | 0 | 0.0% |  | 0 | 0.0% | 0 | 0.0% |  | 0 | 0.0% | 1 | 0.1% |  | 0 | 0.0% | 2 | 0.0% |
| Main symptoms, n (%) | | |  |  |  |  |  |  |  |  |  |  |  |  |  |  |  |  |  |  |  |
|  | Genital symptoms |  | 5,978 | 35.2% | 18,177 | 33.0% |  | 31,749 | 54.9% | 106,596 | 53.0% |  | 370 | 71.7% | 1,211 | 73.3% |  | 5,911 | 80.7% | 16,438 | 80.8% |
|  | Urination-related symptoms |  | 2,336 | 13.7% | 6,890 | 12.5% |  | 5,934 | 10.3% | 16,068 | 8.0% |  | 64 | 12.4% | 137 | 8.3% |  | 616 | 8.4% | 1,380 | 6.8% |
|  | Abdominal Digestive-related symptoms |  | 2,203 | 13.0% | 7,602 | 13.8% |  | 3,957 | 6.8% | 16,181 | 8.0% |  | 3 | 0.6% | 3 | 0.2% |  | 66 | 0.9% | 177 | 0.9% |
|  | Skin and subcutaneous symptoms |  | 2,017 | 11.9% | 6,234 | 11.3% |  | 6,072 | 10.5% | 17,852 | 8.9% |  | 56 | 10.9% | 163 | 9.9% |  | 440 | 6.0% | 1,178 | 5.8% |
|  | Musculoskeletal and joint symptoms |  | 1,974 | 11.6% | 7,777 | 14.1% |  | 4,283 | 7.4% | 18,822 | 9.4% |  | 9 | 1.7% | 82 | 5.0% |  | 151 | 2.1% | 447 | 2.2% |
| Pain score, n (%) | | |  |  |  |  |  |  |  |  |  |  |  |  |  |  |  |  |  |  |  |
|  | 0 |  | 0 | 0.0% | 0 | 0.0% |  | 0 | 0.0% | 0 | 0.0% |  | 0 | 0.0% | 0 | 0.0% |  | 0 | 0.0% | 0 | 0.0% |
|  | 1-3 |  | 2,582 | 15.2% | 10,820 | 19.6% |  | 5,021 | 8.7% | 23,310 | 11.6% |  | 24 | 4.7% | 128 | 7.7% |  | 343 | 4.7% | 1,273 | 6.3% |
|  | 4-6 |  | 2,073 | 12.2% | 7,302 | 13.2% |  | 5,507 | 9.5% | 22,894 | 11.4% |  | 47 | 9.1% | 170 | 10.3% |  | 498 | 6.8% | 1,433 | 7.0% |
|  | 7-10 |  | 784 | 4.6% | 1,941 | 3.5% |  | 2,918 | 5.0% | 7,155 | 3.6% |  | 31 | 6.0% | 48 | 2.9% |  | 253 | 3.5% | 431 | 2.1% |
|  | Not asked |  | 11,567 | 68.0% | 35,070 | 63.6% |  | 44,429 | 76.8% | 147,681 | 73.5% |  | 414 | 80.2% | 1,307 | 79.1% |  | 6,233 | 85.1% | 17,214 | 84.6% |
| Device used to access the website (PC / Mobile), n (%) | | |  |  |  |  |  |  |  |  |  |  |  |  |  |  |  |  |  |  |  |
|  | Mobile |  | 15,058 | 88.5% | 48,836 | 88.6% |  | 56,059 | 96.9% | 195,125 | 97.1% |  | 493 | 95.5% | 1,600 | 96.8% |  | 7,108 | 97.0% | 19,886 | 97.7% |
|  | PC |  | 1,946 | 11.4% | 6,294 | 11.4% |  | 1,814 | 3.1% | 5,906 | 2.9% |  | 23 | 4.5% | 53 | 3.2% |  | 219 | 3.0% | 465 | 2.3% |
|  | unknown |  | 2 | 0.0% | 3 | 0.0% |  | 2 | 0.0% | 9 | 0.0% |  | 0 | 0.0% | 0 | 0.0% |  | 0 | 0.0% | 0 | 0.0% |
| Access to HIV awareness information | | |  |  |  |  |  |  |  |  |  |  |  |  |  |  |  |  |  |  |  |
|  | Not open |  | 13,141 | 77.3% | 43,912 | 79.6% |  | 44,490 | 76.9% | 155,893 | 77.5% |  | 313 | 60.7% | 1,044 | 63.2% |  | 4,413 | 60.2% | 12,717 | 62.5% |
|  | Access to the information page on possible diagnoses | | 3,820 | 22.5% | 11,123 | 20.2% |  | 13,306 | 23.0% | 44,933 | 22.4% |  | 200 | 38.8% | 602 | 36.4% |  | 2,885 | 39.4% | 7,583 | 37.3% |
|  | Access to the information page on HIV testing | | 45 | 0.3% | 98 | 0.2% |  | 79 | 0.1% | 214 | 0.1% |  | 3 | 0.6% | 7 | 0.4% |  | 29 | 0.4% | 51 | 0.3% |
| Survey period | | |  |  |  |  |  |  |  |  |  |  |  |  |  |  |  |  |  |  |  |
|  | October 19, 2022 - December 31, 2022 |  | 3,866 | 22.7% | 12,577 | 22.8% |  | 12,863 | 22.2% | 44,892 | 22.3% |  | 0 | 0.0% | 0 | 0.0% |  | 357 | 4.9% | 952 | 4.7% |
|  | January 1, 2023 - June 30, 2023 |  | 7,168 | 42.1% | 22,862 | 41.5% |  | 24,751 | 42.8% | 83,926 | 41.7% |  | 0 | 0.0% | 0 | 0.0% |  | 823 | 11.2% | 2,343 | 11.5% |
|  | July 1, 2023 - December 27, 2023 |  | 5,972 | 35.1% | 19,694 | 35.7% |  | 20,261 | 35.0% | 72,222 | 35.9% |  | 516 | 100.0% | 1,653 | 100.0% |  | 6,147 | 83.9% | 17,056 | 83.8% |

a; men who have sex with men

b; sexually transmitted infections

Table S4. Positive HIV test results and STI diagnosis in the suggested STI group

| Population | Proportion (%) | Number |
| --- | --- | --- |
| STIs diagnoses among all reported cases | 10.9% | (3037/27821) |
| HIV tested among diagnosed STIs | 9.2% | (280/3037) |
| HIV-positive results among HIV-tested individuals | 4.6% | (13/280) |

Table S5. Intention to undergo post-HIV testing.

|  |  | Total | | Male | | Female | | STIs ^a^ history (+) | |
| --- | --- | --- | --- | --- | --- | --- | --- | --- | --- |
|  |  | n = | 964 | n = | 119 | n = | 838 | n = | 153 |
| Do you know HIV postal testing?, n (%) | |  |  |  |  |  |  |  |  |
|  | Never heard of a self-HIV test | 410 | 42.5% | 51 | 42.9% | 358 | 42.7% | 49 | 32.0% |
|  | Heard of it, but I have not researched it | 122 | 12.7% | 14 | 11.8% | 107 | 12.8% | 17 | 11.1% |
|  | Researched it, but I have not used it | 63 | 6.5% | 7 | 5.9% | 55 | 6.6% | 8 | 5.2% |
|  | Have used it | 15 | 1.6% | 2 | 1.7% | 12 | 1.4% | 2 | 1.3% |
|  | Other | 354 | 36.7% | 45 | 37.8% | 306 | 36.5% | 77 | 50.3% |
| Do you intend to use HIV postal testing from now on? n (%) | |  |  |  |  |  |  |  |  |
|  | Did not have the intention to receive a test, but would be willing to use an HIV postal testing | 180 | 18.7% | 19 | 16.0% | 161 | 19.2% | 19 | 12.4% |
|  | Do not intend to use an HIV postal testing (Have no intention of getting an HIV postal testing) | 88 | 9.1% | 13 | 10.9% | 73 | 8.7% | 12 | 7.8% |
|  | Planning to get an HIV test at a public health center or hospital, so will not use an HIV postal testing | 52 | 5.4% | 8 | 6.7% | 44 | 5.3% | 4 | 2.6% |
|  | Was planning to get an HIV test at a public health center or hospital, but would like to use an HIV postal testing. | 37 | 3.8% | 1 | 0.8% | 36 | 4.3% | 4 | 2.6% |
|  | Originally planned to use an HIV postal testing | 9 | 0.9% | 1 | 0.8% | 7 | 0.8% | 1 | 0.7% |
|  | Other | 598 | 62.0% | 77 | 64.7% | 517 | 61.7% | 113 | 73.9% |

a; Sexually transmitted infections

Figure S1. User flow from symptom assessment to HIV awareness information


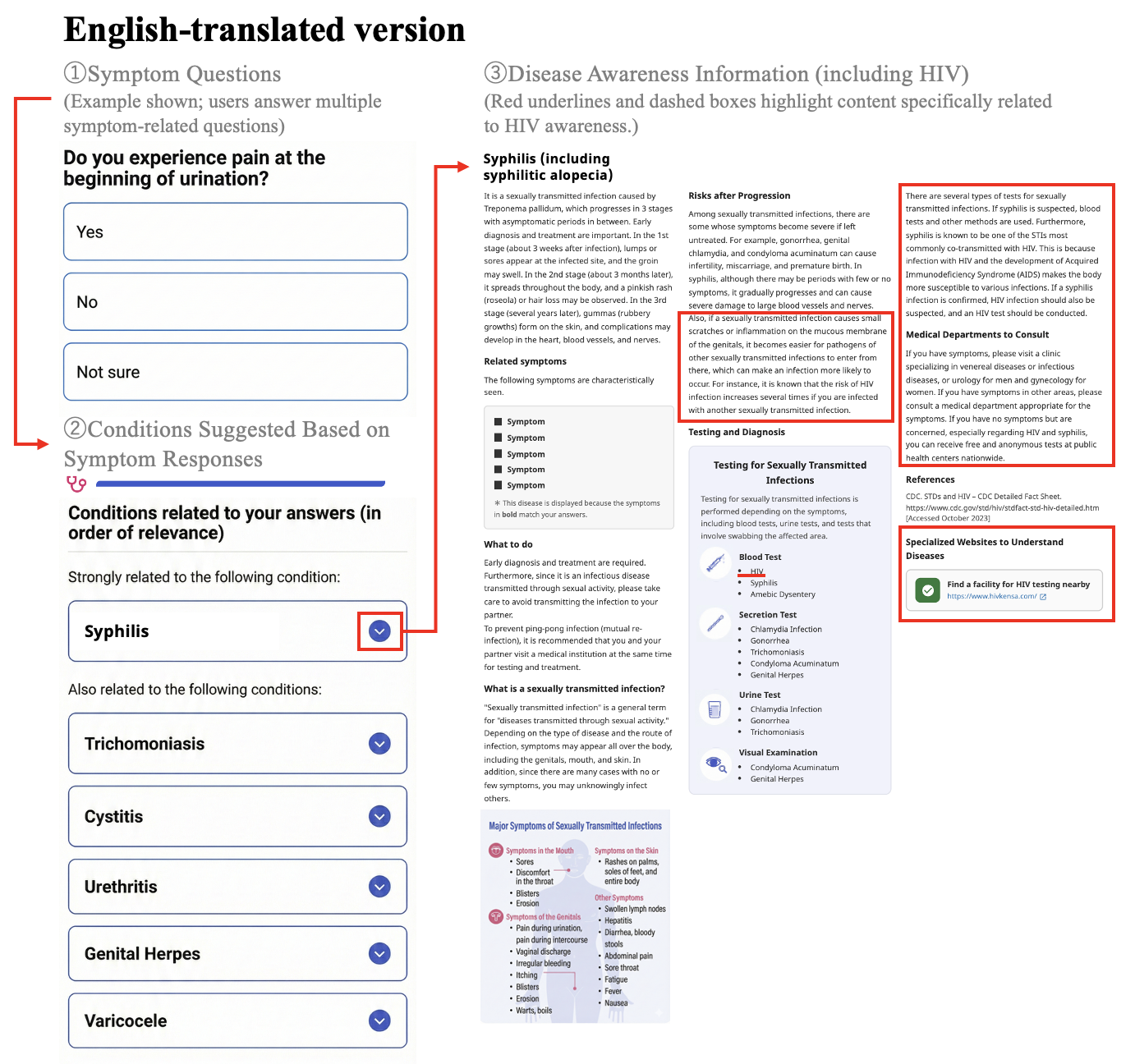


Plain language summary


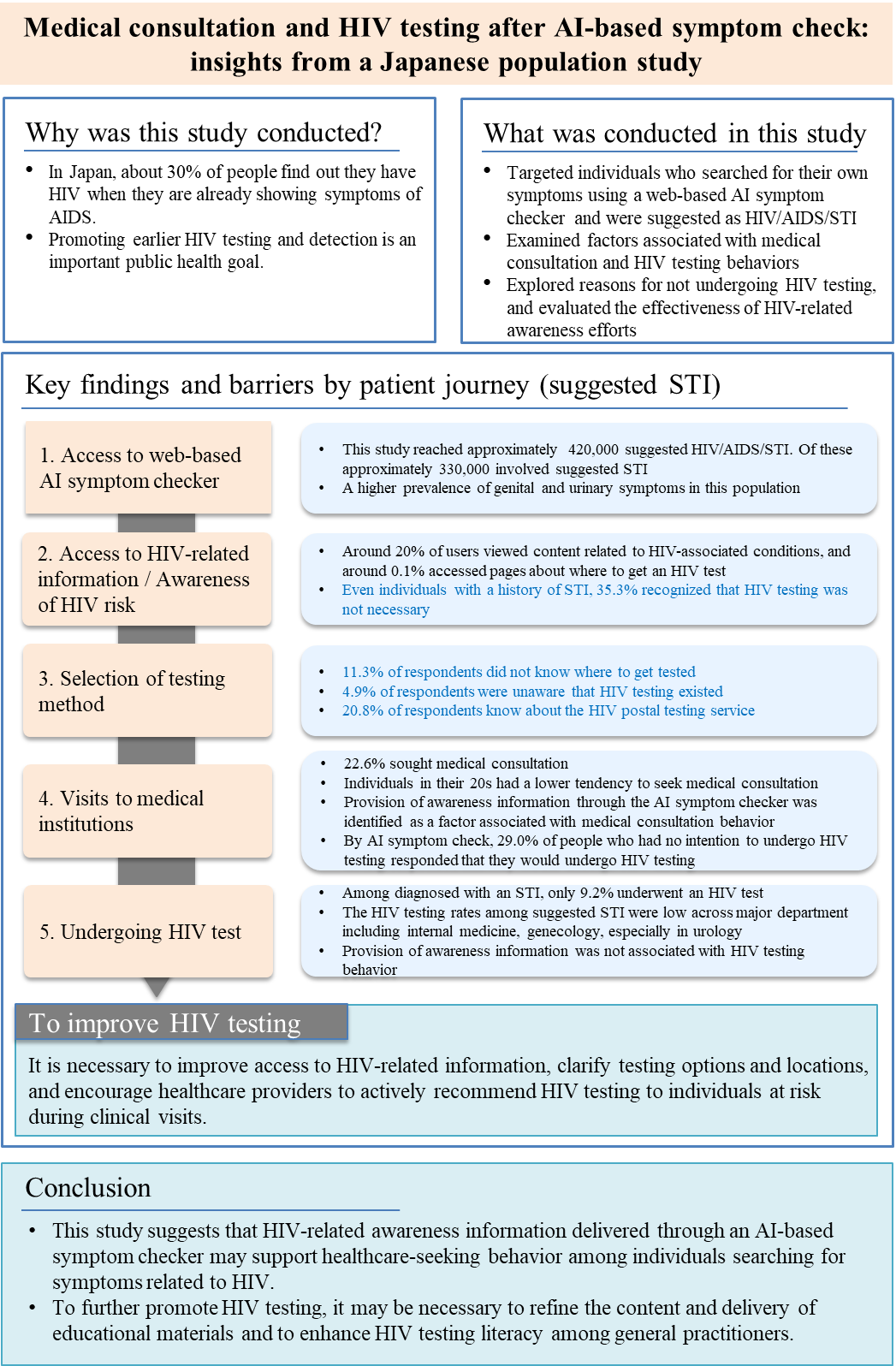

Supplement: Multimedia Appendix 1 [file jmir-v28-e90257-s001.docx]
